# Finite Element Analysis of Generalized Ligament Laxity on the Deterioration of Hallux Valgus Deformity (Bunion)

**DOI:** 10.3389/fbioe.2020.571192

**Published:** 2020-09-08

**Authors:** Duo Wai-Chi Wong, Yan Wang, Tony Lin-Wei Chen, Fei Yan, Yinghu Peng, Qitao Tan, Ming Ni, Aaron Kam-Lun Leung, Ming Zhang

**Affiliations:** ^1^Department of Biomedical Engineering, Faculty of Engineering, The Hong Kong Polytechnic University, Hong Kong, China; ^2^The Hong Kong Polytechnic University Shenzhen Research Institute, Shenzhen, China; ^3^Department of Orthopaedics, Pudong New Area Peoples’ Hospital Affiliated to Shanghai University of Medicine & Health Sciences, Shanghai, China

**Keywords:** metatarsus primus varus, hallux abducto valgus, finite element method, forefoot abduction, pronation

## Abstract

Hallux valgus is a common foot problem affecting nearly one in every four adults. Generalized ligament laxity was proposed as the intrinsic cause or risk factor toward the development of the deformity which was difficult to be investigated by cohort clinical trials. Herein, we aimed to evaluate the isolated influence of generalized ligament laxity on the deterioration using computer simulation (finite element analysis). We reconstructed a computational foot model from a mild hallux valgus participant and conducted a gait analysis to drive the simulation of walking. Through parametric analysis, the stiffness of the ligaments was impoverished at different degrees to resemble different levels of generalized ligament laxity. Our simulation study reported that generalized ligament laxity deteriorated hallux valgus by impairing the load-bearing capacity of the first metatarsal, inducing higher deforming force, moment and malalignment at the first metatarsophalangeal joint. Besides, the deforming moment formed a deteriorating vicious cycle between hallux valgus and forefoot abduction and may result in secondary foot problems, such as flatfoot. However, the metatarsocuneiform joint did not show a worsening trend possibly due to the overriding forefoot abduction. Controlling the deforming load shall be prioritized over the correction of angles to mitigate deterioration or recurrence after surgery.

## Introduction

Hallux valgus is a forefoot deformity characterized by a valgus deviation of the hallux and the widening of the first-second intermetatarsal angle (IMA) (Banks et al., 2001). Exostoses appeared at the dorsomedial side of the first metatarsal head forms into a prominent eminence called bunion in which bursa was further thickened-over predominantly because of the footwear pressure (Perera et al., 2011). The departed hallux and first metatarsal would progress into the subluxation of the first metatarsophalangeal joint (MPJ) and sesamoid apparatus (Banks et al., 2001).

Despite the etiology of hallux valgus remains controversial, the hypermobility or instability of the first ray was recognized as the biomechanical cause of hallux valgus development (Myerson and Badekas, 2000; Deenik et al., 2016). Physicians and researchers hence headed to evaluate the mobility of the first ray by manual examination (Myerson and Badekas, 2000), the Klaue’s device (Klaue et al., 1994), plain radiographs (King and Toolan, 2004), fluoroscopy (Martin et al., 2012), and weight-bearing Computed Tomography (CT) images (Kimura et al., 2017), whilst a meta-analysis has confirmed the association between first ray hypermobility and hallux valgus (Shibuya et al., 2017).

There were several causes of first ray hypermobility proposed. The lesion of the medial supporting structures, including the medial collateral ligament and the medial sesamometatarsal ligament, allowed the first metatarsal head to drift medially and encouraged other unstable movements (Perera et al., 2011). In addition, the deep transverse metatarsal ligament (DTML), a static structural constraint to hypermobility, could prevent the splay of the metatarsals (Stainsby, 1997; Abdalbary et al., 2016). Meanwhile, we supported that the generalized ligament laxity significantly contributed to the development of the hypermobility (McNerney and Johnston, 1979; Glasoe et al., 2010), despite that the prevalence of generalized ligamentous laxity could be as high as 57% in some populations (Hakim and Grahame, 2003; Remvig et al., 2007; Russek and Errico, 2016). Generalized ligament laxity or joint hypermobility is defined as the increase of range of motion across various joints in individuals that associated with musculoskeletal symptoms and risk of injuries (Razak et al., 2014) and is graded using the Beighton score with physical examination (Vallis et al., 2015). A hypermobile foot with reduced ligament stiffness attenuated the directions of joint forces that were suggested to initiate metatarsus primus varus and hallux valgus (Wong et al., 2014b,c). Despite Harris and Beeson (1998) commented that generalized hypermobility was not necessarily a primary predisposing factor to hallux valgus, there was a high incidence of multiple joint laxities among hallux valgus patients (Glasoe et al., 2010), while hypermobility was also linked to the intrinsic causes, such as genetic and sex dimorphism, of hallux valgus (Wilkerson and Mason, 2000; Perera et al., 2011). Furthermore, the manifestation of generalized ligament laxity is not contradictory to the other proposed causes, including the medial supporting structure or DTML failure.

Nevertheless, some literature questioned whether the hypermobility or instability shall be the cause or consequence (Mansur and de Souza Nery, 2020) since a few pieces of evidence were based on cadaveric studies (Uchiyama et al., 2005; Abdalbary et al., 2016) while there is a lack of cohort study to trace the initialization, development, and deterioration of hallux valgus. Implementing cohort studies with clearly defined and isolated exposure factors honed to an unspecific and long time is infeasible since the deterioration factors of hallux valgus could be multifactorial. Computational method using finite element (FE) analysis provides a versatile platform to evaluate the internal biomechanical environment with controlled and pre-assigned sets of conditions (Morales-Orcajo et al., 2016; Wang et al., 2016; Behforootan et al., 2017), which was commonly used to understand foot pathology (Wong et al., 2016, 2018), assess the outcome of surgery (Wang et al., 2019; Wong et al., 2020a), and design implant (Ni et al., 2019; Wong et al., 2020b), etc. The biomechanical consequences of hallux valgus were examined by partial foot models (first ray models) regarding the stress of the medial capsule and the tarsometatarsal and MPJ forces (Wong et al., 2014b; Yu et al., 2020). Moreover, Zhang et al. (2018) predicted the metatarsal stress and pressure of a severe hallux valgus patient under a balanced standing condition using a comprehensive FE model of the foot and ankle complex. On the other hand, using a FE foot model reconstructed from a normal participant, Wong et al. (2014c) predicted the alteration of joint load transfer resulted from the reduction of ligament stiffness and attempted to reveal the potential relationship to hallux valgus. There is a lack of studies that covered research on the potential deterioration factor of hallux valgus.

The objective of this study is to quantify the influence of generalized ligament laxity on the load or stress pattern of the first ray using a computational approach. The effect of generalized ligament laxity was realized by impoverishing the stiffness of ligaments through a parametric analysis. We hypothesized that hallux valgus would be deteriorated by increasing level of laxity which could be attested by the enlargement of deformity angle, reduced load-carrying capacity (joint force and stress) of the first metatarsal, lateralized joint force and abducted moment for the first MPJ and the opposite for the first metatarsocuneiform joint (MCJ).

## Materials and Methods

### Participant Recruitment

**FIGURE 1:**
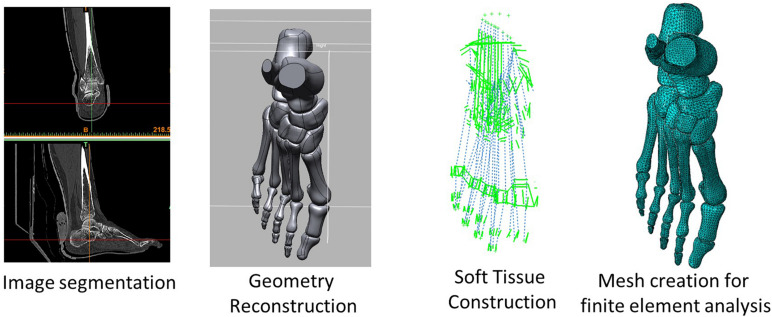
A flow diagram illustrating the process from image acquisition and segmentation, geometry reconstruction, soft tissue reconstruction, and mesh creation for finite element simulation.

A female hallux valgus patient was recruited for the model reconstruction and validation experiment. She was 30 years old, 165 cm in height, and 50 kg weight. The participant’s hallux valgus angle (HVA) and IMA on the transverse plane were 27.2° and 10.4°, respectively, which was classified as a mild level of deformity (Deenik et al., 2016). She had no other apparent hallux valgus related musculoskeletal pathology and previous foot surgery. The process from image digitization, geometry reconstruction to mesh creation for FE simulation is illustrated in Figure 1.

### Geometry Reconstruction

The geometry of the right foot was scanned using CT (Aquilion, Toshiba, Tokyo, Japan). Before the scan, a custom-made ankle-foot orthosis was fabricated to put the ankle joint in a pre-defined neutral position and non-weight-bearing condition as much as possible. The images were scanned with a 0.5 mm interval at 0.831 mm pixel size.

**FIGURE 2:**
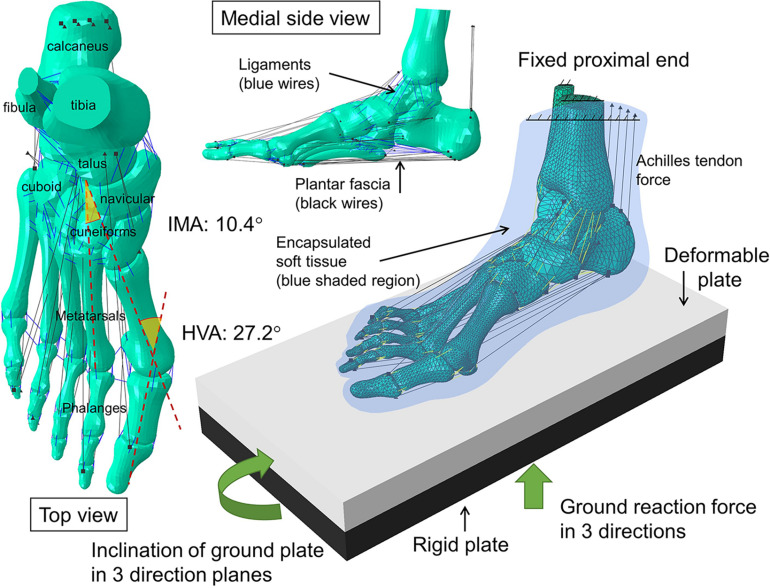
Geometry, and the configuration of boundary and loading conditions of the finite element model of the hallux valgus foot. HVA: hallux valgus angle; IMA: intermetatarsal angle.

The images were segmented and processed in the software, Mimics (Materialise, Leuven, Belgium) and Rapidform XOR2 (3D Systems Korea Inc., Seoul Korea). Thirty bones, including the medial and lateral sesamoids and the encapsulated soft tissue, were modeled as three-dimensional solid parts, as shown in Figure 2. Plantar fascia and ligaments were modeled as slip rings and trusses, respectively. Their locations and configurations were based on the medical images of the model as well as an anatomy atlas (Schuenke et al., 2007) and were subsequently confirmed with an orthopedic surgeon. It shall be noted that the forefoot ligaments were further refined according to Perera et al. (2011), which included the configurations of DTMLs, collateral ligaments, and sesamoid ligaments.

Regarding contacts between model components, the function of the articular cartilage (bone-to-bone contact) was substituted by a non-linear contact stiffness without friction (Athanasiou et al., 1998), whereas the coefficient of friction between the encapsulated soft tissue and the ground was 0.6 (Zhang and Mak, 1999). The interior surface of the encapsulated soft tissue was tied to the adjacent bone surface.

### Mesh Creation

Based on our previous age-matched model that passed the mesh convergence test (Wong et al., 2018), we assumed that the mesh sizes of the encapsulated soft tissue, forefoot bones, and other bones were 4, 2, and 3 mm, respectively. In addition, local refinement was carried out to accommodate contact regions, fine and abrupt geometry. Solid parts including the osseous structures and the encapsulated soft tissue were meshed using the linear tetrahedral elements (C3D4), while trusses were meshed using two-node truss elements (T3D2). The encapsulated soft tissue was wrapped by a layer of 3-node triangular membrane elements (M3D3) to represent the skin layer.

The mesh creation process was conducted in the FE software, Abaqus 6.11 (Dassault Systèmes, Vélizy-Villacoublay, France). The number of elements for the bones, the encapsulated soft tissue, and the skin layer was 130,836, 157,214, and 7,526, respectively.

### Material Property

The material properties used in the FE model is listed in Table 1. The assigned materials were assumed homogeneous in all parts and were assumed linearly elastic for the osseous structures (Chen et al., 2003). Hyperelastic material properties were assigned to the encapsulated soft tissue (Lemmon et al., 1997) and the skin layer using the second-order polynomial strain energy potential equation and the first-order Ogden model (Gu et al., 2010). The second-order polynomial strain energy potential equation was adopted with the form

$$U=\sum_{i+j=1}^{2}C_{i\,j}\,{\left({\bar{I}}_{1}-3\right)}^{i}\,{\left({\bar{I}}_{2}-3\right)}^{j}+\sum_{i=1}^{2}\frac{1}{D_{i}}\,{\left(J_{e\,1}-1\right)}^{2\,i}$$

**TABLE 1:** Material properties used in the finite element model.

Part	Material property	References
Bone	*E* = 10 GPa, ν = 0.3	Chen et al., 2003
Encapsulated soft tissue	Hyperelastic (second-order polynomial strain energy potential equation, *C*_10_ = 0.08556 Nmm^–2^, *C*_01_ = −0.05841 Nmm^–2^, *C*_20_ = 0.03900 Nmm^–2^, *C*_11_ = −0.02319 Nmm^–2^, *C*_02_ = 0.00851 Nmm^–2^, *D*_1_ = 3.65273 mm^2^N^–1^, *D*_2_ = 0.0000 mm^2^N^–1^)	Lemmon et al., 1997
Ground plate (deformable layer)	*E* = 10,000 GPa, ν = 0.1	
Ligament	*E* = 260 MPa, ν = 0.4, Cx-A = 18.4 mm^2^	Siegler et al., 1988
Plantar fascia	*k* = 203.3 Nmm^–1^ (1st column) *k* = 232.5 Nmm^–1^ (2–4th column) *k* = 182.2 Nmm^–1^ (5th column)	Kitaoka et al., 1994
Skin	Hyperelastic (first-order Ogden model, μ = 0.122 MPa, α = 18), *T* = 2.0 mm	Gu et al., 2010

*Cx-A: Cross-sectional area; *E*: Young’s modulus; *k*: elastic stiffness; *T*: Thickness; ν: Poisson’s ratio.*

where *U* is the strain energy per unit of reference volume; *C*_*ij*_ and *D*_*i*_ are material coefficients in Table 1; *Î*_1_ and *Î*_2_ are the first and second deviatoric strain invariants defined as

$${\bar{I}}_{1}={\bar{\lambda }}_{1}^{2}+{\bar{\lambda }}_{2}^{2}+{\bar{\lambda }}_{3}^{2}$$

$${\bar{I}}_{2}={\bar{\lambda }}_{1}^{\left(-2\right)}+{\bar{\lambda }}_{2}^{\left(-2\right)}+{\bar{\lambda }}_{3}^{\left(-2\right)}$$

with the deviatoric stretches ${\bar{\lambda }}_{i}=J_{e\,1}^{-1/3}\,\lambda_{i}\cdot J_{e\,1}$ and λ*_*i*_* are the elastic volume ratio and the principal stretches, respectively.

The first-order Ogden model was represented in the form of

$$U=\frac{2\,\mu }{\alpha^{2}}\,\left(\lambda_{1}^{\alpha }+\lambda_{2}^{\alpha }+\lambda_{3}^{\alpha }-3\right)$$

where *U* is the strain energy per unit reference volume; λ_1_, λ_2_, and λ_3_ are the deviatoric principal stretches; α and μ are material coefficients in Table 1.

Cross-sectional area information was supplemented to the ligament truss, while specific stiffness on different columns was assigned to the plantar fascia that modeled using slip ring connectors to resemble the windlass mechanism (Siegler et al., 1988; Kitaoka et al., 1994). The Young’s modulus of the ground plate was assigned with a large value to mimic a rigid ground.

### Boundary and Loading Condition

The model participant was asked to perform five repeated walking trials over-ground at a self-selected comfortable speed in a 10-m gait laboratory to acquire the boundary and loading conditions for the FE simulation. The laboratory was equipped with an eight infra-red based camera motion capture system (Mx-40, Vicon, Oxford Metrics Ltd., Oxford, United Kingdom) and force platforms (AMTI, Watertown, MA, United States), which operated at 200 and 1000 Hz, respectively. Before the experiment, infra-red reflective markers were attached to the body of the model participant that complied with the marker set of the musculoskeletal model (John et al., 2013).

The kinematics and kinetics data from the gait experiment was exported to the musculoskeletal model platform (OpenSim version 3.3, National Center for Simulation in Rehabilitation Research, Stanford, United States) to predict the muscle forces during the walking trials (Delp et al., 2007). The generic model, gait-2392, was scaled according to the mass and body dimension of the model participant (John et al., 2013). The process of inverse kinematics/dynamics was implemented in which the dynamic inconsistency was optimized by adjusting the model pass properties via the residual reduction algorithm module. Muscle forces were then estimated by the built-in computed muscle control modules. The estimated muscle force during walking stance included the gastrocnemius-and-soleus complex (via tendon Achilles), tibialis anterior, tibialis posterior, extensor digitorum longus, extensor hallucis longus, flexor digitorum longus, flexor hallucis longus, fibularis brevis, and fibularis longus.

**FIGURE 3:**
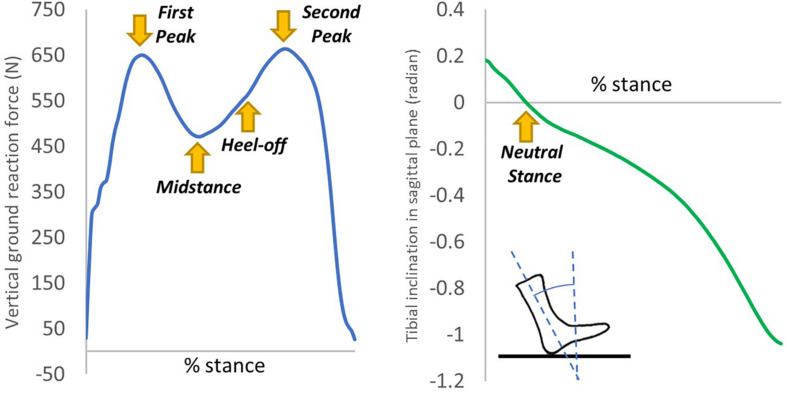
An illustration of the extraction of the six instants from the vertical ground reaction force and tibial inclination in the sagittal plane of one typical trial.

The boundary and loading conditions were extracted from featured instants according to the vertical ground reaction force (GRF) and the tibial inclination to ground in the sagittal plane since a quasi-static approach was implemented in the FE simulation. The instants were heel-strike (∼0% stance), neutral stance (∼14% stance), GRF first peak (∼20% stance), midstance (∼40% stance), heel-off (∼55% stance), GRF second peak (∼70% stance). The exact stance percentage of the featured instants varied from trial to trial depending on the ankle joint position and the vertical GRF. The heel-strike instant started with a vertical GRF larger than 10 N. As shown in Figure 3, the neutral stance happened when the tibial was vertical to the ground. The GRF first peak, midstance, and second peak were defined as the first peak, valley, and second peak points of the vertical GRF graph, while the heel-off instant was assumed the midpoint between the midstance and the GRF second peak. The proximal ends of the tibia and fibula were fixed, while the GRFs in the three directions and the shank-to-ground angles in the three plane directions were applied on the ground plate, as shown in Figure 2. Muscle forces were applied through concentric load along with the corresponding muscle directions, except the tendon Achilles that the muscle force was applied through concentrated force superiorly.

### Model Output and Analysis

The FE simulation was conducted in the commercial FE package Abaqus 6.11 (Dassault Systèmes, Vélizy-Villacoublay, France) using a general implicit (quasi-static) solver. A parametric analysis was conducted with five different levels (conditions) of generalized ligament laxity in which the stiffness of the ligaments was reduced by none (intact), 20, 40, 60, and 80%. For each condition, five sets of simulations were carried out using the boundary and loading conditions from the data of the five walking trials acquired from the gait experiments and the musculoskeletal models.

Outcome measures included the maximum von Mises stress of the metatarsal shafts, the angulations, joint forces, and moments of the first ray. The maximum von Mises stress of the metatarsal bone shafts indicated the attenuated load transfer pattern upon different levels of general laxity, whereas the angulations (HVA and IMA), joint forces, and joint moments in the coronal plane of the first ray reflected the strength of deterioration of hallux valgus. We measured the angulation by exporting the corresponding bone meshes at each instant after simulation and generated their axes based on a regression of the mesh coordinates that operated in Rapidform XOR2 (3D Systems Korea Inc., Seoul Korea). The joint forces and moments were the FE predicted contact forces and moments across the articular joints (Wang et al., 2018). Data at the heel-strike and neutral stance instants were not shown since the changes of outcome were negligible before the forefoot was loaded, but the simulations of these instants were essential since the loading history may influence the outcome of the simulated instants afterward.

### Statistical Analysis

We considered the walking trial data (simulation schemes) as the independent observations to implement inferential statistics as suggested by existing literature (Chen and Chen, 2014; Araujo et al., 2016) to deal with the single-subject design (N-of-1 trials) in this study. However, it shall be noted that the interpretation of the statistical analysis or probability values was confined to the internal validity accounting for the variation of the boundary and loading conditions among walking trials.

All statistical analysis was performed in SPSS 21 (IBM, New York, NY, United States). Before the statistical analysis, the Shapiro–Wilk test was performed to check for data normality. To determine whether there were any statistical differences among the levels of generalized ligament laxity, one-way ANOVA repeated measures were performed for data with normality satisfied; otherwise, a Friedman test was performed (*n* = 5). If a significant main effect was found, *post hoc* pairwise comparison was followed using a Bonferroni adjusted *p*-value. The Greenhouse–Geisser correction was implemented on all data because of their violation of sphericity. The level of significance was set at *p* = 0.05, two-tailed, and was marked on the condition with a higher level of laxity in the pairwise comparison in the Tables. All data were expressed as mean (standard deviation) and the effect size was evaluated using partial eta square (η_*p*_^2^) unless otherwise specified.

### Validation

Model validation was conducted by comparing the peak pressures of dedicated regions between the measurements and FE predictions. A plantar pressure measurement system (F-scan system, Tekscan, South Boston, MA, United States) was equipped by the model participant during the gait experiment. The plantar area of the result data was divided into seven regions, including the heel, midfoot, medial metatarsal, central metatarsal, lateral metatarsal, hallux, and lesser toes regions, in which the maximum pressure of each region during GRF first peak, midstance, heel-off, and GRF second peak, were extracted for analysis unless the value was zero. To eliminate the initialization and termination effects, the first and the last walking trial data were discarded. The extracted data of the three successive walking steps were paired to that of the FE prediction in intact condition (0% laxity). Eventually, there were 33 sets of non-zero maximum pressure data pairs between the measurement and FE prediction (*n* = 33).

The evaluation of agreement for validation was conducted using correlation analysis and the Bland-Altman plot (Henninger et al., 2010) which was carried out using GraphPad Prism 8.4.2 (GraphPad Software, San Diego, CA, United States). The Pearson correlation |r| value of ≤0.35 is considered weak correlation, 0.36–0.67 moderate correlations, and 0.68–0.9 strong correlation (Taylor, 1990).

## Results

### Validation

**FIGURE 4:**
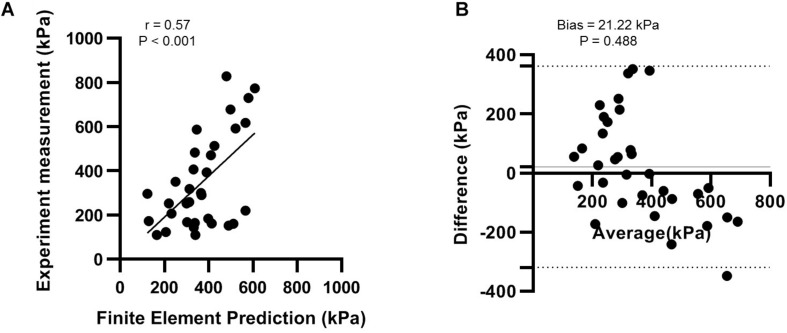
Validation of finite element model with experimental measurement using **(A)** correlation analysis; and **(B)** the Bland-Altman plot.

As shown in Figure 4, the correlation analysis indicated that there was a significant moderate linear relationship between the measurement and FE prediction (*r* = 0.573 95% CI 0.285 to 0.765; *p* < 0.001). The Bland-Altman plot showed that there was an insignificant mean offset of 21.22 kPa (*p* = 0.488). The deviation could be because the sensor redistributed the plantar pressure slightly (Wong et al., 2018). Based on these results, we viewed our FE simulation validated and adequately reliable.

### Angulation and Alignment

**FIGURE 5:**
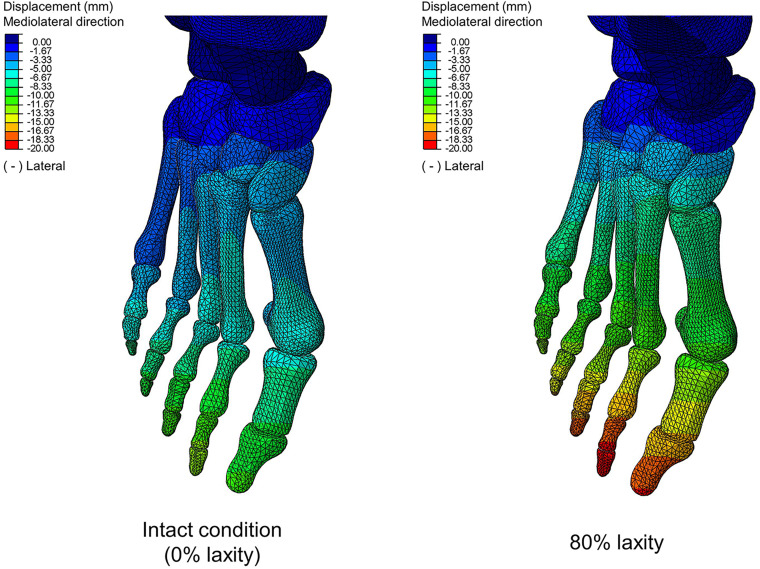
Bone displacement between intact and 80% laxity conditions in mediolateral direction at second peak instant. Negative values denote lateral direction.

Table 2 shows that there were significant differences in HVA at the first peak (χ^2^(4) = 17.120; *p* = 0.002; Kendall’s *W* = 0.856) and second peak instants (*F*(1.991, 7.965) = 4.547; *p* = 0.048; η_*p*_^2^ = 0.532). From intact to 80% laxity, HVA decreased by 2.3% and increased by 1.1%, at the first and second peak instants, respectively. Similarly, significant differences were also found in IMA at the first peak (χ^2^(4) = 15.200; *p* = 0.004; Kendall’s *W* = 0.760), midstance (χ^2^(4) = 20.000; *p* < 0.001; Kendall’s *W* = 1.000) and second peak instants (*F*(1.409, 5.635) = 20.654; *p* = 0.003; η_*p*_^2^ = 0.838). IMA at 80% laxity was significantly increased compared to all other levels of laxity at the second peak instant (*p* < 0.05). Figure 5 illustrates the displacement of the metatarsal bones in the mediolateral direction and show an apparent forefoot abduction by observation.

**TABLE 2:** Hallux valgus angle (HVA), 1-2 intermetatarsal angle (IMA), and the joint force directions of the first metatarsocuneiform joint (MCJ) and the first metatarsophalangeal joint (MPJ) among different levels of generalized ligament laxity during stance.

Instant	Laxity level					
	Intact (0%)	20%	40%	60%	80%	*p*-value	Effect size
Hallux valgus angle (°), HVA							
First Peak	32.8 ± 0.84	33.02 ± 0.79	32.67 ± 1.03	32.5 ± 1.04	32.06 ± 0.94	**0.002^†^**	0.856
Midstance	30.93 ± 0.58	30.87 ± 0.57	30.95 ± 0.68	30.81 ± 0.75	30.42±0.67)(3	0.173	0.399
Heel-off	31.82 ± 0.82	31.93 ± 0.63	32.06 ± 0.45	32.05 ± 0.54	32.08 ± 0.83	0.674	0.051
Second Peak	32.88 ± 0.63	32.73 ± 0.73	32.87 ± 0.67	32.93 ± 0.73	33.23 ± 0.87	**0.048**	0.532
1-2 intermetatarsal angle (°), IMA							
First Peak	13.65 ± 0.29	13.74 ± 0.31	13.82 ± 0.34	13.93 ± 0.39	14.11 ± 0.52	**0.004^†^**	0.760
Midstance	12.65 ± 0.37	12.69 ± 0.34	12.73 ± 0.31	12.79 ± 0.29	12.92 ± 0.29	**<0.001^†^**	1.000
Heel-off	11.99 ± 0.19	11.98 ± 0.18	12.01±0.19)(2	12.14 ± 0.25	12.37 ± 0.42	0.088	0.559
Second Peak	11.6 ± 0.31	11.57 ± 0.22	11.56 ± 0.15	11.7±0.21)(2	11.97±0.25)(1)(2)(3)(4	**0.003**	0.838
Direction of metatarsocuneiform joint (MCJ) force (°)							
First Peak	15.77 ± 1.78	15.42 ± 1.91	14.95 ± 2.06	14.2±2.15)(1)(2)(3	13.01±2.23)(1)(2)(3)(4	**0.001**	0.945
Midstance	12.37 ± 2.44	12.02 ± 2.49	11.56±2.54)(2	10.87±2.58)(1)(2)(3	9.76±2.74)(1)(2)(3)(4	**0.001**	0.941
Heel-off	11.03 ± 0.78	10.72 ± 0.77	10.25 ± 0.81	9.48±0.93)(2)(3	8.08±1.23)(1)(2)(3)(4	**0.003**	0.914
Second Peak	10.12 ± 0.76	9.54±0.84)(1	8.62±1.01)(1)(2	7.34±1.04)(1)(2)(3	6.43 ± 2.06	**0.008**	0.831
Direction of metatarsophalangeal joint (MPJ) force (°)							
First Peak	−7.75 ± 0.47	−8.35 ± 0.52	-9.24±0.59)(2	-10.67±0.71)(1)(2)(3	-13.41±1.04)(1)(2)(3)(4	**0.001**	0.944
Midstance	−4.55 ± 2.55	−5.14 ± 2.51	−6 ± 2.43	−7.39 ± 2.32	−10.1 ± 2.24	0.058	0.630
Heel-off	−0.95 ± 1.72	−1.55 ± 1.8	−2.5 ± 1.89	−4.19 ± 1.98	−7.82 ± 2.07	**<0.001^†^**	1.000
Second Peak	0.61 ± 1.03	−0.1 ± 1.03	−1.24 ± 1.06	−3.2 ± 0.98	−6.07 ± 1.77	**0.023**	0.719
Maximum von Mises stress (MPa)							
First Peak	3.35 ± 0.82	3.4 ± 0.84	3.44 ± 0.86	3.48 ± 0.85	3.53 ± 0.83	**0.004**	0.804
Midstance	06 ± 0.61	4.09 ± 0.61	4.11 ± 0.6	4.14 ± 0.59	4.07 ± 0.54	0.230	0.332
Heel-off	7.42 ± 1.59	7.44 ± 1.58	7.41 ± 1.56	7.23 ± 1.48	6.64 ± 1.27^(3)(4)^	**0.006**	0.873
Second Peak	14.22 ± 2.43	13.95 ± 2.2	13.55 ± 1.98	12.87 ± 1.95	11.54 ± 2.68^(1)(2)^	**0.002**	0.912

*Positive values on the joint force direction denote angle from the anterior axis toward the medial direction. Bold font indicates a significant difference, two-tailed (*p* < 0.05) in one-way ANOVA repeated measure or Friedman test. Significance difference, two-tailed (adjusted *p* < 0.05) in *post hoc* pairwise test compared to ^(1)^ intact condition; ^(2)^ 20% level of laxity; ^(3)^ 40% level of laxity; and ^(4)^ 60% level of laxity. ^†^Statistics analysis conducted using Friedman test because of the data non-normality with an effect size using Kendall’s W.*

### Stress of the Metatarsal Shaft

**FIGURE 6:**
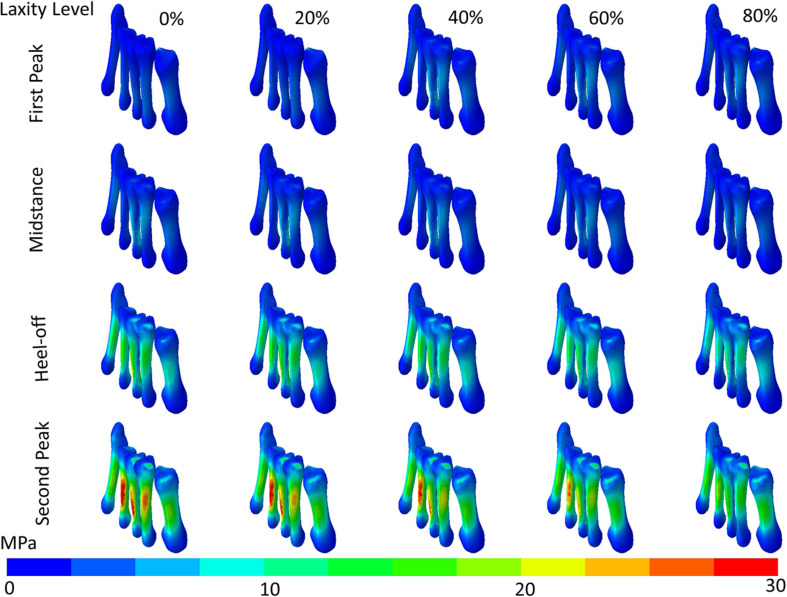
von Mises stress distribution (contour plot without deformation and displacement) of the metatarsal shafts in different levels of generalized laxity at different stance instants in one typical set of simulation.

**FIGURE 7:**
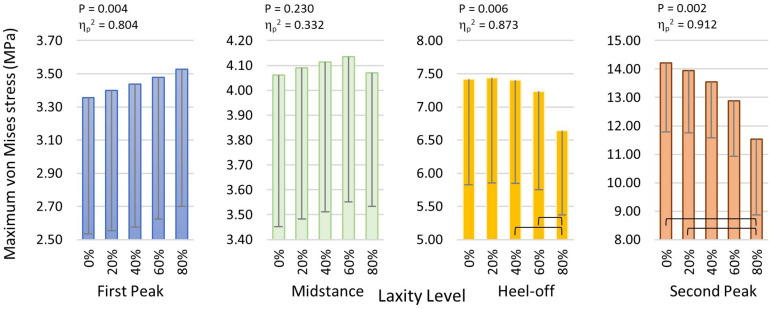
Maximum von Mises stress of the first metatarsal shaft in different laxity levels during stance. Brackets between pair denote significant difference in *post hoc* pairwise comparison (adjusted *p* < 0.05). Error bars indicate standard deviation.

The maximum von Mises stress of the metatarsal shafts are shown in Figure 6. The stress elevated as the stance progressed but declined when the laxity level advanced. In Figure 7, there were significant differences in the maximum von Mises stress of the first metatarsal shaft among the levels of laxity at the first peak (*F*(1.570, 6.279) = 16.40; *p* = 0.004; η_*p*_^2^ = 0.804), heel-off (*F*(1.015, 4.062) = 27.536; *p* = 0.006; η_*p*_^2^ = 0.873), and second peak (*F*(1.105, 4.420) = 41.331; *p* = 0.002; η_*p*_^2^ = 0.912) instants. When the level of laxity advanced to 80%, the stress was significantly decreased compared to that of the intact condition (mean difference = 2.679 MPa, 95% CI 1.676 to 3.681; *p* = 0.001) and 20% at second peak instant (mean difference = 2.409 MPa, 95% CI 0.882 to 3.935; *p* = 0.009); and was also significantly decreased compared to that of the 40% (mean difference = 0.762 MPa, 95% CI 0.009 to 1.514; *p* = 0.048) and 60% at the heel-off instant (mean difference = 0.590 MPa, 95% CI 0.042 to 1.138; *p* < 0.05).

### Joint Force and Moment of the First MCJ and MPJ

**FIGURE 8:**
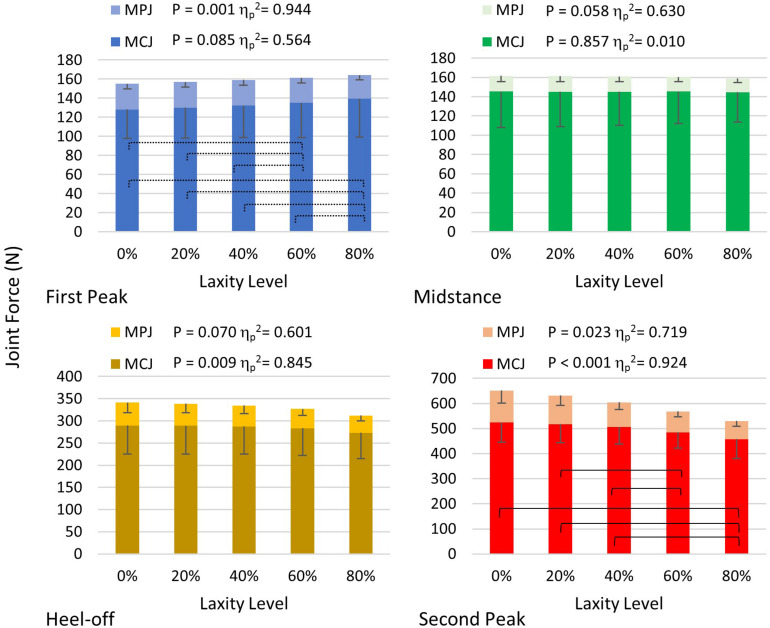
First MCJ and MPJ force in different levels of laxity during stance. Brackets between pair in the graph of second peak denote significant difference in *post hoc* pairwise comparison in MCJ (adjusted *p* < 0.05); stroked brackets in the graph of first peak between pair denote significant difference in *post hoc* pairwise comparison in MPJ (adjusted *p* < 0.05). Error bars indicate standard deviation.

As shown in Figure 8, the first MC joint force was significantly reduced at heel-off (*F*(1.021, 4.083) = 21.889; *p* = 0.009; η_*p*_^2^ = 0.845) and second peak (*F*(1.619, 6.475) = 48.546; *p* < 0.001; η_*p*_^2^ = 0.924) instants. Particularly at the second peak instant, the joint force of the 80% laxity condition was significantly lower than that of the intact condition (mean difference = 66.240 N, 95% CI 35.947 to 96.533; *p* = 0.003), 20% condition (mean difference = 59.512 N, 95% CI 29.092 to 89.931; *p* = 0.004), and 40% condition mean difference = 48.095 N, 95% CI 9.093 to 87.098; *p* = 0.023). Regarding the MPJ force, there were significant differences at the first and second peak instants, whereas the *post hoc* comparison demonstrated that the MPJ force at 80% level of laxity was significantly smaller than the other laxity conditions at first peak instant (*p* < 0.05).

The joint force directions of the first MCJ were significantly lateralized at all instants (*p* < 0.05), whereas that of the first MPJ were significantly lateralized at first peak (*F*(1.038, 4.154) = 237.342; *p* < 0.001; η_*p*_^2^ = 0.983) and second peak instants (*F*(1.070, 4.282) = 49.912; *p* = 0.002; η_*p*_^2^ = 0.926). With a laxity level of 80%, the first MCJ and MPJ directions were lateralized by 3.7° and 6.9°, respectively, compared to that of the intact condition (Table 2).

All first MCJ and MPJ showed significant increases of abduction moments (*p* < 0.005), except for that of the MPJ at the second peak instant in which significance was not demonstrated, as shown in Table 3. At heel-off instants, the first MCJ and MPJ abduction moments significantly increased by 13.9% (mean difference = 1371.623 N mm, 95% CI 290.242 to 2453.004; *p* = 0.021) and 43.8% (165.073 N mm, 95% CI 41.491 to 288.655; *p* = 0.017), respectively from intact to 80% level of laxity condition.

**TABLE 3:** Joint moment of the first metatarsocuneiform joint (MCJ) and the first metatarsophalangeal joint (MPJ) among different levels of generalized ligament laxity during stance.

Instant	Laxity level					
	Intact (0%)	20%	40%	60%	80%	*p*-value	Effect size
Joint moment of the first metatarsocuneiform joint, MCJ (N mm)							
First Peak	−3469.78 ± 989.43	−3618.22 ± 1086.76	−3822.82 ± 1221.73	−4120.42 ± 1407.43	−4630.95 ± 1710.42	**0.026**	0.750
Midstance	−4229.51 ± 942.08	−4345.87 ± 947.08^(1)^	−4504.26 ± 956.67^(1)(2)^	−4759.89 ± 969.23^(1)(2)(3)^	−5153.8 ± 981.6^(1)(2)(3)(4)^	**<0.001**	0.981
Heel-off	−9189.82 ± 2226.06	−9409.56 ± 2287.98^(1)^	−9678.7 ± 2375.75^(1)(2)^	−10051.95 ± 2485.12^(1)(2)(3)^	−10561.45 ± 2594.16^(1)(2)(3)(4)^	**0.002**	0.924
Second Peak	−20320.81 ± 4763.62	−20789.87 ± 4814.76	−21399.1 ± 4901.61^(1)(2)^	−21923.39 ± 4793.45	−21523.84 ± 4845.88	**0.009**	0.738
Joint moment of the first metatarsophalangeal joint, MPJ (N mm)							
First Peak	−513.2 ± 131.83	−556.58 ± 147.49^(1)^	−614.65 ± 168.62^(1)(2)^	−695.21 ± 193.21^(1)(2)(3)^	−797.65 ± 221.9^(1)(2)(3)(4)^	**0.002**	0.924
Midstance	−214.3 ± 34.34	−237.85 ± 38.72^(1)^	−269.74 ± 45.22^(1)(2)^	−315.6 ± 51.76^(1)(2)(3)^	−379.37 ± 65.21^(1)(2)(3)(4)^	**0.001**	0.933
Heel-off	−643.82 ± 275.65	−690.37 ± 288.58^(1)^	−750.47 ± 303.73^(1)(2)^	−841.95 ± 320.02^(1)(2)(3)^	−1004.83 ± 344.01^(1)(2)(3)(4)^	**<0.001**	0.963
Second Peak	−2701.38 ± 1683.01	−2676.78 ± 1524.84	−2650.5 ± 1342.08	−2598.64 ± 1096	−2439.18 ± 717.4	0.610	0.072

*Negative values indicate abduction moment. Bold font indicates a significant difference, two-tailed (*p* < 0.05) in one-way ANOVA repeated measure or Friedman test. Significance difference, two-tailed, (adjusted *p* < 0.05) in *post hoc* pairwise test compared to ^(1)^ intact condition; ^(2)^ 20% level of laxity; ^(3)^ 40% level of laxity; and ^(4)^ 60% level of laxity.*

## Discussion

Hallux valgus (or bunion) is one of the most prevalent musculoskeletal problems affecting 23% of the adults and 35.7% of the elderly population (Nix et al., 2010). It reduces the quality of life, impairs balance and gait, and increases falling risks (Munteanu et al., 2017). Genetic and sex dimorphisms are the intrinsic causes and undoubtedly predisposes general laxity and hypermobility that plays a major role in driving the development of hallux valgus (Mansur and de Souza Nery, 2020). This study contributed by suggesting the deteriorating trend to help direct surgical assessment and decision (Mansur and de Souza Nery, 2020), or design orthotic treatment, whereas physicians can assess the level of generalized ligament laxity or hypermobility of patients through simple clinical tests.

The first ray of the foot is one of the main load-bearing structures and key components to maintain the medial longitudinal arch yet has not been designed to be more stable (Perera et al., 2011). It has been the subject of debate regarding the function of the first tarsometatarsal joint and its relationship to the etiology of hallux valgus (Doty and Coughlin, 2013). There is no tendon attachment on the first metatarsal head. The stability of the first metatarsophalangeal and tarsometatarsal joints highly relies on the dynamic restraints contributed by the extrinsic muscles, tibialis anterior, and fibularis longus, especially during propulsion when both stability and flexibility are demanding (Cornwall et al., 2006; Perera et al., 2011). When the ligamentous tissue goes laxity, it fails to secure the first ray from the excessive dorsal excursion and therefore extends the degree and duration of pronation during push-off. The excessive and prolonged pronation undermines the leverage of the extrinsic muscles and weakened their functions of stability and effective propulsion that further exacerbates the hypermobility (Cornwall et al., 2006). Hypermobility implicates other foot pathologies, such as acquired flatfoot, posterior tibial tendon dysfunctions, and plantar fasciitis that also in turn further deteriorates hallux valgus deformity (Cornwall et al., 2006). On the other hand, the medial divergence on the developed deformity elicits the bow-string effect on the distally tethered plantar fascia and flexor hallucis longus which advance the deforming force (Stainsby, 1997), causing a vicious cycle.

Our FE predictions provided partial support for our initial hypothesis. The stress of the first metatarsal bone and the joint force of the first MCJ were significantly decreased during late stance in cases of the generalized ligament laxity. The weakened windlass mechanism contributed by the deformity impaired the load-carrying capacity of the first metatarsal, which was also substantiated by the lower plantar pressure and shear of the medial forefoot in some studies though inconsistent (Yavuz et al., 2009; Nix et al., 2013; Hida et al., 2017). In addition, Zhang et al. (2018) simulated a balanced standing condition on a severe hallux valgus participant and found a significantly lower metatarsal joint load. Although higher stress was also found on the metatarsals, the stress was concentrated at the joint regions which could be caused by joint incongruency of the deformity. Besides, in our study, the lateral deviation of the 1st phalanx at the MPJ (hallux abducto valgus) was further exaggerated with an increasing level of laxity which was demonstrated by significant enlargement of HVA and lateralized MPJ force during push-off and abducted moment during load acceptance phase.

**FIGURE 9:**
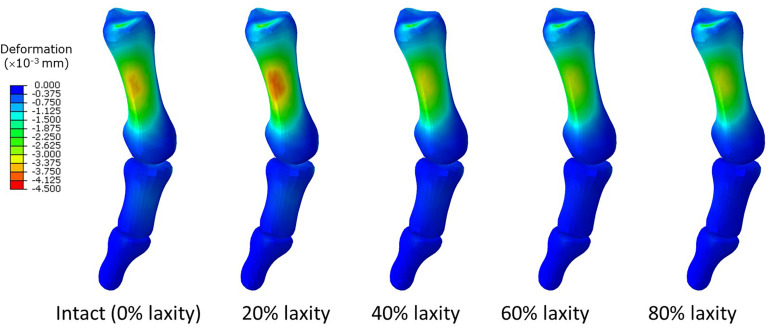
Deformation of the first metatarsal, proximal and distal phalanx in different laxity levels in second peak instant. Negative value denotes compressive deformation.

In contrast, our study could not provide sufficient evidence relating generalized ligament laxity to the medial deviation of the first metatarsal (metatarsus primus varus) for the progression of hallux valgus. The reason could be an apparent abduction of the forefoot, which may override the influence of metatarsus primus varus and potentially couple with pronation and arch collapse. As shown in Figure 5, the difference of mediolateral displacement between the intact and 20% laxity condition was 6.84 mm at the second peak instant. In addition, the deformation of the first metatarsal was neither sensitive nor a presented linear trend with respect to the change of ligament laxity. The maximum compressive principal strain of the first metatarsal at the second peak instant was varied in the range between 0.142 and 0.115% over different levels of laxity. As illustrated in the representative simulation scheme in Figure 9, the maximum magnitude of the compressive deformation ranged from 3.19 × 10^–3^ mm to 4.32 × 10^–3^ mm and 0.48 × 10^–3^ mm to 0.54 × 10^–3^ mm, respectively for the first metatarsal and the hallux, in comparison to the mesh size of 2 mm. In fact, the high degree of abduction moments at both MCJ and MPJ could exaggerate the forefoot abduction problem. Although some studies proposed that medialized load was generated by ligament laxity that triggered metatarsus primus varus and thus hallux valgus (Wong et al., 2014b,c), they targeted on the initialization process using a normal foot model without deformity and may not implicate the progression process.

We believed that the biomechanical cause of the hallux valgus is intrinsic. The impairment of either static or dynamic stabilizers, or misalignment induced by other deformities, shall be the root of the problem (Perera et al., 2011). Extrinsic factors, such as wearing high-heeled shoes, could increase the intensity and exposure of the high and unbalanced joint moments during gait that speeds up the problem (Cornwall et al., 2006). Although our simulation did not show a large magnitude of deforming angle or load, it shall be noted that the deformity problem is accumulative and is subjected to time and exposure. This could be the reason why hallux valgus is prevalent in the elderly population but age is not a good predictor of hallux valgus (Perera et al., 2011).

According to the pathogenesis of the hallux valgus proposed by Perera et al. (2011), the medial deviation of the first metatarsal came before the lateral deviation of the 1st phalanx. Therefore, some surgical interventions aimed to stabilize the first metatarsal using different techniques (Wong et al., 2014a; ElAttar et al., 2018). Our study found that the manifestation of the first metatarsal medial deviation induced by deforming load may be hindered by the forefoot abduction or pronation that formed a viscous cycle to provoke more deforming load. Controlling the pronation or eversion by supporting the medial longitudinal arch by orthosis (Glasoe et al., 2010; Nakagawa et al., 2019; Peng et al., 2020) may slow down the deterioration process and supplement surgical outcomes to reduce recurrence.

Simulations of surgeries for hallux valgus were also conducted using the FE method. Wong et al. (2015) attempted to explain the complication of the Lapidus procedure (the 1st MCJ fusion) and found that there was high tensile stress located at the bone graft inferiorly that could lead to non-union of the fusion. Guo et al. (2018) found that distal metatarsal osteotomy could reduce the sesamoid displacement and redistributed the plantar pressure whereas the team also compared bandaging, Kirschner fixation, and fiberglass fixation in their later study (Mao et al., 2017). Besides, Brilakis et al. (2019) compared the procedure variation of Mitchell’s procedure with and without pins and quantified the additional stability contributed by the use of pins. However, none of these simulations could address the high recurrence in surgical treatment (Lucattelli et al., 2020) and we hoped that our study regarding the relationship between intrinsic cause and deterioration factors could provide more insights to tackle this issue.

There were some weaknesses in this study. The simplifications and assumptions inherited to the geometry segmentation and reconstruction, materials, and loading conditions were inevitable in simulation research. For example, the majority of the foot models simplified the bones to be homogeneous without the segmentation of the trabecular core and cortical layer because of the complexity, size, and number of the bones involved in the whole foot and ankle structure (Morales-Orcajo et al., 2016). The assumption may affect the strain of the metatarsals (Trabelsi et al., 2014). Besides, we assumed that the GRFs and the shank-to-ground angle profile were not significantly different during the course of the parametric test. In addition, intrinsic muscles may play a major role in dynamic stabilization but were not considered in our study due to the lacking of data (Nix et al., 2013). Measuring EMG signals of the leg muscles could improve the accuracy of muscle force estimation using the musculoskeletal model and thus the accuracy of the input for the FE model (Chen et al., 2019).

The single-subject design can eliminate the main effect of patient variation and is efficient to investigate treatment outcome (Araujo et al., 2016). However, there were debates on whether inferential statistics could be applied to the single-subject design (Toothaker et al., 1983; Araujo et al., 2016). In addition, the lack of external validity by the single-subject design impacts the translation of the evidence into practice in which studies with sufficient sample size are now considered the *sine qua non*. In fact, research involving FE foot models often adopted a single-subject design that overlooked external validity because of the strenuous work involved. It remains difficult and impractical to reconstruct a few complex foot-and-ankle models with gait experiments and validation for each model participant (Morales-Orcajo et al., 2016; Wang et al., 2016). Instead, internal validity was taken into account by the evaluation of inter-trial variance in the model analysis and validation (Chen et al., 2020). We also aimed to select a model participant with a typical physique and characteristics of the foot pathology to compromise external validity. Comparing current findings to the FE model predictions of a control participant could provide additional insights into this study. Future work may investigate the different levels of hallux valgus severity and their interactions among HVA and IMA, or to explore other intrinsic causes of hallux valgus, such as the lesion of medial ligaments or DTML. The simulation of extrinsic causes and its interactions with intrinsic causes could uncover the role of high-heeled shoes in the development of hallux valgus (Yu et al., 2013, 2016).

## Conclusion

In conclusion, we reported that a higher level of generalized ligament laxity deteriorated hallux valgus by reducing the joint load and stress of the first metatarsal which implicates a failed load-carrying capacity of the first ray. The deterioration of the hallux valgus happened distally at the first MPJ with a higher deforming force, moment, and alignment, whereas the proximal side (the first MCJ) did not show a worsening trend and could probably due to the overriding forefoot abduction. The high abduction moment for both first MCJ and MPJ may exaggerate the forefoot abduction and subsequent pronation. Orthotic or other conservative interventions may help alleviate the deforming force and moment in order to mitigate deterioration or recurrence after surgery. Current model could be improved by further segmentation of the trabecular core and cortical layer. Future work can consider different levels of hallux valgus severity and explore other intrinsic causes of hallux valgus, such as the lesion of medial ligaments or DTML.

## Data Availability Statement

All datasets presented in this study are included in the article/Supplementary Material.

## Ethics Statement

The studies involving human participants were reviewed and approved by The Human Subjects Ethics Sub-committee of the Hong Kong Polytechnic University. The patients/participants provided their written informed consent to participate in this study.

## Author Contributions

DW, AL, and MZ planned the studies. MN and AL facilitated patient recruitment, the CT scan, and medical consultation. DW, YW, and TC reconstructed the model and conducted the finite element analysis. YW, TC, and YP conducted the gait experiment and analysis. TC and FY conducted the muscle force estimation in the musculoskeletal model. QT handled the data processing and checking. DW conducted the data and statistical analysis, and wrote the manuscript. MZ obtained funding and supervised the project. All authors contributed to revising and editing the manuscript.

## Conflict of Interest

The authors declare that the research was conducted in the absence of any commercial or financial relationships that could be construed as a potential conflict of interest.
